# Pulmonary Benign Metastasizing Leiomyoma in a Patient With a Concurrent Breast Tumor: A Diagnostic Challenge

**DOI:** 10.7759/cureus.96392

**Published:** 2025-11-08

**Authors:** Tomoyuki Araya, Toshiyuki Kita, Takayuki Higashi, Ryo Hara, Hazuki Takato

**Affiliations:** 1 Respiratory Medicine, National Hospital Organization (NHO) Kanazawa Medical Center, Kanazawa, JPN

**Keywords:** 18f fdg-pet/ct, benign metastasizing leiomyoma (bml), differential diagnosis, estrogen receptor (er), multiple pulmonary nodules

## Abstract

Pulmonary benign metastasizing leiomyoma (PBML) is a rare condition in which histologically benign smooth muscle tumors occur in distant organs, most often the lungs. Although typically discovered incidentally, differentiating BML from metastatic malignancy can be challenging in real-world clinical settings. A 53-year-old woman with a history of hysterectomy for uterine leiomyoma presented with multiple pulmonary nodules and a cystic left breast lesion containing an irregular solid component. Fluorine-18 fluorodeoxyglucose positron emission tomography/computed tomography demonstrated no significant fluorodeoxyglucose (FDG) uptake in the pulmonary nodules but intense uptake in the cystic lesion of the left breast. Excisional biopsy of the breast revealed an intraductal papilloma, while video-assisted thoracoscopic biopsy of the lung nodules demonstrated benign smooth muscle proliferation positive for α-smooth muscle actin and estrogen receptor (ER), with a very low Ki-67 index, findings consistent with BML. This case illustrates the diagnostic challenge of distinguishing BML from metastatic malignancy in patients with concurrent lesions. Awareness of this entity and its characteristic low FDG uptake may help clinicians avoid unnecessary overtreatment and adopt an appropriate diagnostic strategy.

## Introduction

Benign metastasizing leiomyoma (BML) represents a paradoxical condition in which histologically benign uterine-type smooth muscle proliferations appear at distant sites, most commonly in the lungs [1,2]. It primarily affects women with a prior history of uterine leiomyoma or hysterectomy, and fewer than 200 cases have been reported to date [3]. Because the radiologic features, typically multiple, well-circumscribed pulmonary nodules, often mimic metastatic carcinoma, accurate diagnosis depends on histologic confirmation and careful clinicopathologic correlation [3,4].

Although the precise pathogenesis remains debated, two major mechanisms have been proposed: hematogenous spread of benign uterine smooth muscle cells after surgery and hormone-dependent proliferation of ectopic smooth muscle cells [5,6]. Understanding these mechanisms is important for differentiating BML from malignant metastasis.

On 18F-fluorodeoxyglucose positron emission tomography/computed tomography (FDG-PET/CT), pulmonary BML (PBML) lesions generally exhibit absent or low FDG uptake, with a median maximum standardized uptake value (SUVmax) of approximately 2.2, and about 90% of reported lesions showing low accumulation [7]. Recognition of this imaging characteristic may help differentiate BML from malignant metastases and prevent unnecessary systemic therapy.

We report a case of PBML identified during the evaluation of multiple lung nodules in a woman with a concurrent breast lesion. This case underscores that PBML should be considered only after malignancy has been rigorously excluded in women presenting with multiple pulmonary nodules, particularly when other lesions coexist.

## Case presentation

A 53-year-old woman was referred for evaluation of abnormal chest imaging showing multiple pulmonary nodules. She had undergone a total hysterectomy with bilateral salpingectomy for uterine leiomyoma eight years earlier, with both ovaries preserved. The patient was completely asymptomatic, with no cough, dyspnea, chest pain, or systemic symptoms. Physical examination was unremarkable. Laboratory findings, including complete blood count, liver and renal function tests, and tumor markers (carcinoembryonic antigen, cytokeratin 19 fragment, pro-gastrin-releasing peptide, carbohydrate antigen 19-9, cancer antigen 125, and soluble interleukin-2 receptor) were all within normal limits (Table 1).

**Table 1:** Laboratory findings at presentation WBC: white blood cells; Neut: neutrophils; Lymph: lymphocytes; Mono: monocytes; Eos: eosinophils; Baso: basophils; RBC: red blood cells; Hb: hemoglobin; Ht: hematocrit; Plt: platelets; CRP: C-reactive protein; T-Bil: total bilirubin; TP: total protein; ALP: alkaline phosphatase; AST: aspartate aminotransferase; ALT: alanine aminotransferase; LDH: lactate dehydrogenase; Alb: albumin; Na: sodium; K: potassium; Cl: chloride; BUN: blood urea nitrogen; Cre: creatinine; eGFR: estimated glomerular filtration rate; UA: uric acid; CEA: carcinoembryonic antigen; CYFRA 21-1: cytokeratin 19 fragment; Pro-GRP: pro-gastrin-releasing peptide; CA19-9: carbohydrate antigen 19-9; CA125: cancer antigen 125; sIL-2R: soluble interleukin-2 receptor; β-D-glucan: beta-D-glucan; Asp Ag: *Aspergillus *antigen; Crypto Ag: cryptococcal antigen; IGRA: interferon-γ release assay; anti-GPL IgA: anti-glycopeptidolipid core IgA antibody; ANCA: anti-neutrophil cytoplasmic antibody Comprehensive laboratory test results at the time of initial evaluation. All parameters were within their respective reference ranges except for a mildly elevated D-dimer level, which was clinically nonspecific

Parameter (unit)	Result	Reference range
WBC (/µL)	4500	4500-9000
Neut (%)	63.9	38-74
Lymph (%)	17.4	16.5-49.5
Mono (%)	7.9	5-10
Eos (%)	9.9	0-10
Baso (%)	0	0-2
RBC (×10⁴/µL)	442	382-500
Hb (g/dL)	13.7	11.7-14.6
Ht (%)	41.1	34.3-44.2
Plt (×10⁴/µL)	25.4	13.0-37.0
CRP (mg/dL)	0.02	0-0.4
T-Bil (mg/dL)	0.5	0.3-1.2
TP (g/dL)	7.1	6.7-8.3
ALP (U/L)	82	38-113
AST (U/L)	13	13-33
ALT (U/L)	5	6-27
LDH (U/L)	166	119-229
Alb (g/dL)	4.3	4.0-5.0
Na (mEq/L)	141	135-149
K (mEq/L)	3.7	3.5-4.9
Cl (mEq/L)	105	96-108
BUN (mg/dL)	9.7	8-22
Cre (mg/dL)	0.54	0.5-0.8
eGFR (mL/min/L)	90.0	60-100
UA (mg/dL)	3.7	2.3-7.0
HbA1c (%)	5.7	<6.4
D-dimer (µg/mL)	1.1	0-1
CEA (ng/mL)	1.7	<3.5
CYFRA 21-1 (ng/mL)	1.2	<3.5
Pro-GRP (pg/mL)	29.8	<81
CA19-9 (U/mL)	2.1	<35.0
CA125 (U/mL)	10.9	<35.0
sIL-2R (U/mL)	203	157-474
β-D-glucan (pg/mL)	3.714	<11.0
Asp Ag	0.0	<0.5
Crypto Ag	Negative	Negative
IGRA	Negative	Negative
Anti-GPL IgA	Negative	Negative
Myeloperoxidase-ANCA (U/mL)	<1.0	<3.5
Proteinase 3-ANCA (U/mL)	<1.0	<3.5

**Figure 1:**
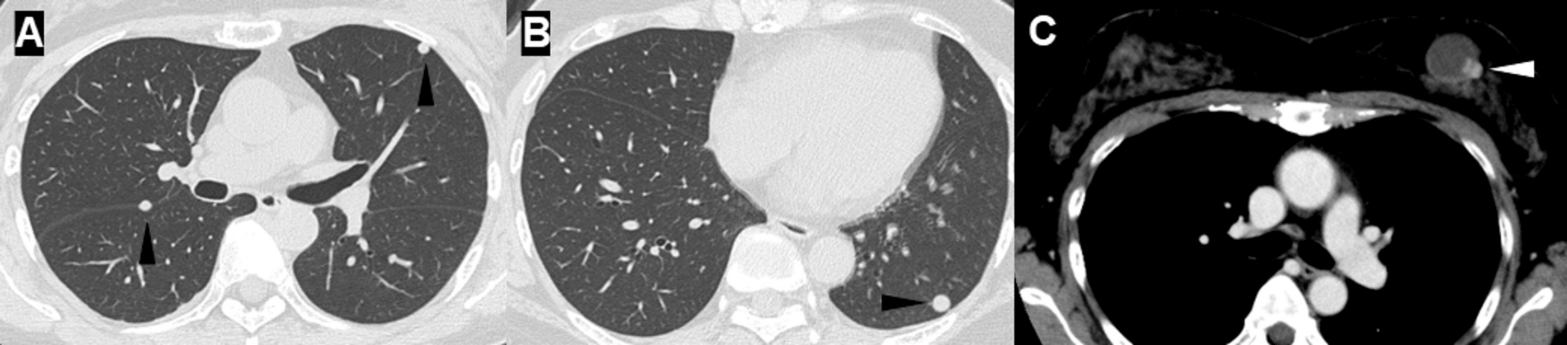
Chest and breast imaging findings on computed tomography Chest computed tomography showing multiple, well-defined nodules in both lungs (A, B; arrowheads) and a cystic lesion with a solid component in the left breast (C; arrowhead)

Tests for infectious diseases, including *Mycobacterium tuberculosis*, nontuberculous mycobacteria, and fungal infections (β-D-glucan, *Aspergillus *antigen, and cryptococcal antigen), as well as for autoimmune diseases (myeloperoxidase- and proteinase 3-antineutrophil cytoplasmic antibodies), were all negative (Table 1). Chest CT revealed multiple, well-defined nodules in both lungs without pleural effusion or lymphadenopathy (Figures 1A, 1B). In addition, a cystic lesion with an irregular solid component was detected in the left breast (Figure 1C).

**Figure 2:**
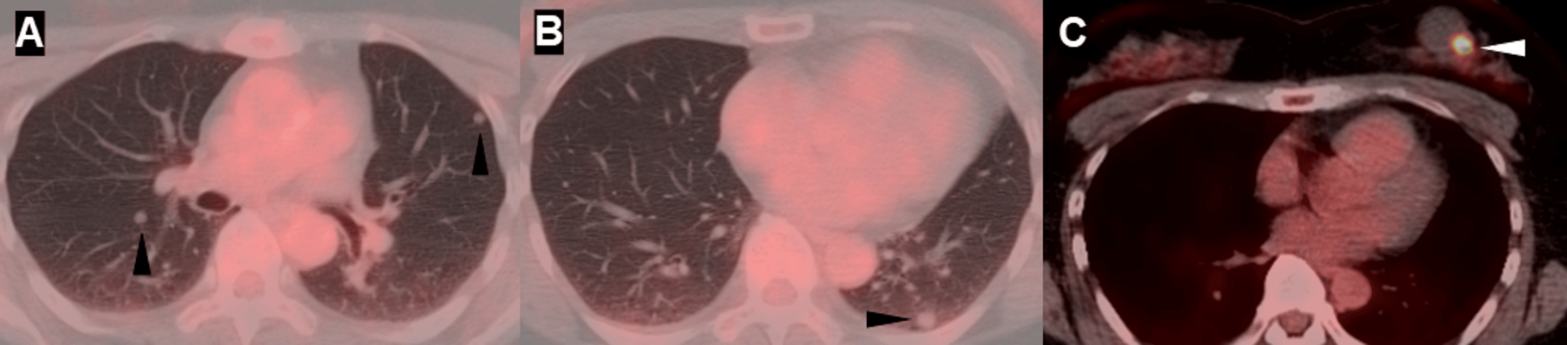
Fluorodeoxyglucose positron emission tomography/computed tomography findings of pulmonary nodules and breast lesion Fluorodeoxyglucose positron emission tomography/computed tomography demonstrating no significant fluorodeoxyglucose uptake in multiple pulmonary nodules (A, B; arrowheads) but intense fluorodeoxyglucose accumulation with a maximum standardized uptake value of 8.9 in a cystic lesion of the left breast (C; arrowhead)

FDG-PET/CT demonstrated no significant 18F-FDG uptake in the pulmonary nodules but showed intense accumulation (SUVmax = 8.9) corresponding to the cystic breast lesion (Figure 2).

**Figure 3:**
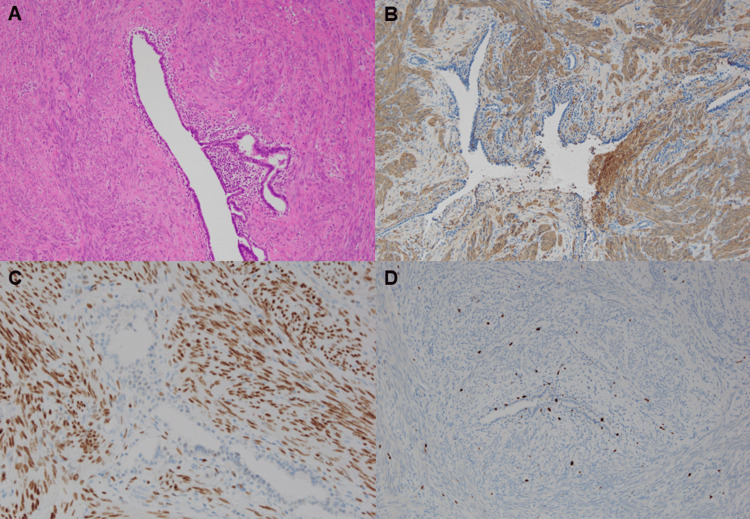
Histopathological and immunohistochemical findings of the lung nodule (A) Hematoxylin and eosin staining showing bundles of benign smooth muscle cells surrounding bronchioles (×100). (B) Tumor cells diffusely positive for α-smooth muscle actin (×100). (C) Strong nuclear positivity for estrogen receptor (×200). (D) Low proliferative activity demonstrated by MIB-1 (Ki-67) immunostaining (×100)

Given the patient’s family history of breast cancer, pulmonary metastases from breast carcinoma were initially suspected. However, excisional biopsy of the breast lesion revealed an intraductal papilloma. To establish a definitive diagnosis, video-assisted thoracoscopic (VATS) wedge resection of two pulmonary nodules was performed. Histopathological examination demonstrated interlacing bundles of bland spindle cells lacking atypia or mitotic activity (Figure 3A). Immunohistochemistry showed positivity for α-smooth muscle actin and estrogen receptor (ER) (Figures 3B, 3C), with a very low Ki-67 index (Figure 3D), confirming the diagnosis of PBML.

Considering the patient’s asymptomatic status and postmenopausal condition, conservative management with periodic surveillance was chosen, and the residual lesions have remained unchanged without the appearance of any new lesions during 15 months of follow-up.

## Discussion

This case highlights the diagnostic complexity of PBML in patients presenting with concurrent lesions suspicious for malignancy. Although BML is histologically benign, its radiologic presentation as multiple pulmonary nodules often mimics metastatic disease, making misdiagnosis a genuine concern. Therefore, imaging findings must always be corroborated by histologic confirmation before assuming malignancy.

In this patient, the coexistence of a PET-positive breast lesion strongly suggested metastatic breast cancer. However, the striking discordance between intense FDG uptake in the breast lesion and absent uptake in the pulmonary nodules prompted reconsideration of alternative diagnoses. Several reports have documented that BML lesions may demonstrate only faint or non-avid FDG uptake on PET/CT, aiding differentiation from malignant lesions. For example, Nakajo et al. described a case of multiple PBML nodules with minimal FDG uptake [8], and Kao et al. reported a patient with concurrent breast cancer whose pulmonary nodules exhibited low FDG accumulation, raising suspicion of BML in the differential diagnosis [9]. In a broader review, Sawai et al. summarized 36 PBML cases, noting that approximately 90% showed low or absent FDG uptake [7]. Collectively, these findings suggest that low metabolic activity on PET/CT, while not diagnostic, serves as an important clue distinguishing BML from metastatic disease.

The decision to perform a VATS biopsy was pivotal. In patients with known or suspected malignancy, histologic confirmation of pulmonary lesions remains essential before assuming metastatic spread, as treatment strategies differ profoundly. Several reviews emphasize that the exclusion of malignancy is a critical diagnostic step when evaluating pulmonary nodules in women with a history of uterine leiomyoma [4].

Pathogenetically, two principal mechanisms have been proposed for PBML: (i) hematogenous dissemination of benign uterine smooth muscle cells, often following uterine surgery, and (ii) hormonally driven extra-uterine proliferation, including coelomic metaplasia. The former is widely regarded as the predominant mechanism, supported by reports of vascular invasion following uterine procedures [5] and by clinicopathologic analyses demonstrating frequent ER/progesterone receptor (PR) expression and low proliferative indices in pulmonary lesions [6]. Conversely, the latter mechanism is supported by histopathologic evidence of coelomic rests and diffuse hormone-receptor positivity in extra-uterine foci [3].

In our patient, en bloc hysterectomy without morcellation argues against iatrogenic intravascular seeding, whereas strong ER positivity with preserved ovarian function favors a hormone-dependent mechanism. Moreover, molecular studies demonstrating clonal relatedness between uterine and extra-uterine lesions support a uterine origin in many cases, although emerging evidence suggests etiologic heterogeneity and possible alternative pathways [3].

Ultimately, this case underscores that PBML should be considered in the differential diagnosis only after malignancy has been meticulously excluded, particularly in women with concurrent or suspicious lesions. Given that fewer than 200 cases have been reported worldwide, BML remains an exceptional entity whose diagnosis should never precede thorough exclusion of malignant disease. From a practical standpoint, even when imaging or histologic features favor BML, clinicians must approach management with caution when coexisting lesions raise concern for malignancy. Integrating imaging findings, recognizing the limitations of PET/CT, and performing selective tissue sampling are essential to prevent both misdiagnosis and overtreatment.

## Conclusions

Although imaging findings such as multiple, well-defined pulmonary nodules with low FDG uptake may suggest a benign process, malignancy must always be rigorously excluded when other lesions raise suspicion. This case emphasizes the importance of a cautious, evidence-based diagnostic approach that integrates imaging and histologic evaluation to ensure appropriate management and avoid both overtreatment and false reassurance. In addition, clinicians should recognize that PBML can mimic metastatic disease both radiologically and clinically, particularly in women with prior uterine surgery. Long-term follow-up is essential because the biological behavior of BML remains unpredictable, and new lesions may emerge even after years of stability. Awareness of this rare entity can help improve diagnostic accuracy and optimize patient care in similar complex presentations.
